# French women's representations and experiences of the post‐treatment management of breast cancer and their perception of the general practitioner's role in follow‐up care: A qualitative study

**DOI:** 10.1111/hex.12518

**Published:** 2016-11-29

**Authors:** Catherine Laporte, Julie Vaure, Anne Bottet, Bénédicte Eschalier, Clémentine Raineau, Denis Pezet, Philippe Vorilhon

**Affiliations:** ^1^ Department of General Medicine Faculty of Medicine of Clermont‐Ferrand Université d'Auvergne Clermont‐Ferrand France; ^2^ EA 7280 NPsy‐Sydo University of Auvergne Clermont‐Ferrand France; ^3^ Maison des Sciences de l'Homme Blaise Pascal University Clermont‐Ferrand France; ^4^ Service de Chirurgie Digestive Unité d'Oncologie—UMR Unité Inserm/Université d'Auvergne U1071 University Hospital Clermont‐Ferrand—Hôpital d'Estaing Clermont‐Ferrand France; ^5^ PEPRADE (Périnatalité, grossesse, Environnement, PRAtiques médicales et DEveloppement) Clermont Université Université d'Auvergne Clermont‐Ferrand France

**Keywords:** breast cancer survivors, general practitioner, post‐treatment follow‐up, qualitative research

## Abstract

**Background:**

In France, the Cancer Plan II 2009‐2013 was launched to improve post‐cancer management and promote greater involvement of general practitioners (GPs) in follow‐up care.

**Objectives:**

We investigated how women experienced the post‐treatment management of breast cancer and perceived the role of the GP in follow‐up care.

**Design:**

We conducted a qualitative study based on semi‐structured interviews with women with breast cancer in remission. The interviews were transcribed and analysed in accordance with the principles of thematic analysis.

**Setting and participants:**

We interviewed 21 patients aged between 30 and 86. Eighteen breast cancer survivors were recruited from GP practices and five from a patients’ association.

**Results:**

Four themes emerged from the thematic analysis: that breast cancer is a life‐changing event; how patients managed the effects of treatment; how patients viewed the future; and patients’ expectations of their GP.

**Discussion and conclusion:**

French survivors of breast cancer perceived the physical changes caused by their illness to impact their womanhood, leading to difficulties with sexual relations, a diminished sense of self and fears for the future. They felt abandoned at the end of treatment and desired support. They appreciated the ease of contacting their GP but considered follow‐up care outside their remit. They agreed to be followed up by their GP, provided that they co‐operated closely with a cancer specialist. This is in accordance with the French Cancer Plan II 2009‐2013, which recommends greater involvement of GPs in a monitoring protocol shared with cancer specialists.

## 1 Introduction

Because of its high incidence and relatively high survival rate, breast cancer is the most prevalent cancer in the general population.^1^, ^2^, ^3^ In France, all women with breast cancer are entitled to initial therapeutic management tailored to their individual case and to the effects of their treatment. They receive care in a hospital setting in accordance with standard therapeutic regimens. The last session of chemo‐ or radiotherapy marks the beginning of “life after breast cancer.”^4^ Screening for relapse is an essential part of follow‐up, but it is not the only wish of survivors, who would welcome greater overall coordination.^5^

Many studies have examined the psychosocial impact and late effects of breast cancer on women.^6^ Women treated for breast cancer describe their life as radically altered and feel that they must create a new life after cancer.^7^ They report that it is difficult to settle back into family life and reconcile their new life with work and social commitments. When their treatment ends, their subsequent management is not individualized and they regret receiving no psychosocial support.^8^ In France, studies have investigated the quality of life of breast cancer survivors,^9^ but few have focused on their experiences, needs and the challenges they face upon the completion of hospital treatment.

Because women have easy access to their general practitioner (GP), the most recent international recommendations^10^, ^11^ suggest that family doctors should participate in the post‐hospital follow‐up of patients who have finished treatment. In setting up the Cancer Plan II 2009‐2013, the French government aimed to individualize the management of patients and increase the involvement of GPs, both during and after treatment.^12^ The *Institut National du Cancer* (French National Cancer Institute) and *Haute Autorité de Santé* (National Authority for Health), while recognizing the value of GPs’ contributions to cancer management, called for the development of new strategies to improve their role in post‐cancer treatment.^13^ Primary care physicians play an increasing role in monitoring the evolution and the late and long‐term effects of the disease, while the involvement of oncologists in follow‐up decreases.^14^, ^15^ Several studies have shown that women think that GPs lack experience with cancer and have insufficient time, but patients are not opposed to the involvement of GPs in follow‐up care under the condition of good coordination with an oncologist.^15^, ^16^, ^17^ Given the implementation of the Cancer Plan II 2009‐2013, it seemed relevant to collect the views of French survivors of breast cancer on their post‐treatment follow‐up. Therefore, in this study, we examined how French women experienced the post‐treatment management of breast cancer and perceived the role of the GP in follow‐up care.

## 2 Methods

### 2.1 Study design

We used a qualitative method modelled on an exploratory approach appropriate for recording perceptions. This exploratory approach was based on individual semi‐structured interviews that examined the perceptions and experiences related to private and painful life events of breast cancer survivors.^18^ We adhered to the *Consolidated Criteria for Reporting Qualitative Studies* guidelines.^19^

To collect a greater diversity of experiences, women were recruited from the practice populations of GPs in the Auvergne region of central France and from the patients’ association of a regional cancer centre. The GPs of this population were identified because they met variability criteria in terms of sex, environment (urban, semi‐rural or rural) and type of practice (group or individual). Of 16 GPs selected from the telephone directory, nine agreed to participate. The president of the patients’ association and the GPs were trained in how to recruit patients for the study by JV. To be eligible, patients had to be over 18 years of age and experiencing their first episode of breast cancer, the treatment for which (surgery, radio‐ or chemotherapy but not hormone therapy) had to have terminated more than 1 month and fewer than 5 years earlier. Patients were excluded if they had a relapsing form of breast cancer, cognitive disorders diagnosed by the GP or severe deterioration of their general state of health (eg because of another form of cancer, chronic heart failure or respiratory failure). The patients were chosen to obtain a purposive sample according to descriptive (age, residence, occupation, family situation before and after cancer, number of children) and strategic (type of treatment, time since the end of institutional treatment, membership of a patients’ association) variables. The number of interviews was determined by the principle of data saturation, which in this study was defined by a lack of new themes raised during three consecutive interviews.

The president of the patients’ association and the GPs invited eligible women to participate in the study and obtained their consent. If a patient declined to participate, the reason was recorded. Interviews were conducted by the same researcher (JV) through face‐to‐face, semi‐structured conversations. All interviews took place in the patients’ homes. The interviewer introduced herself as a medical student conducting a research project on the post‐treatment management of breast cancer. The interview guide (Table 1) was designed on the basis of reports from the literature,^17^, ^20^, ^21^, ^22^, ^23^ French^10^ and international recommendations^10^, ^24^ and discussions with a public health anthropologist (CR) working at the cancer centre. Initially, three pilot interviews were conducted to allow the interviewer to adjust to the interview guide. During the interviews, patients were questioned about the psychological and intimate aspects of post‐treatment life events and how they affected their job, the effects of their treatment and the role of their GP in their follow‐up care. Following the pilot interviews, we added a question about patients’ experiences of cancer treatment.

**Table 1:** Interview guide

Tell me about your breast cancer
When you were diagnosed with cancer what questions did you ask yourself about the future?
How did you cope with your treatment? What role did your attending physician play during treatment?
And now, after treatment, how are things, what are your feelings?
What has the cancer changed in your personal life?
How was the programme of medical supervision of your cancer organised?
Explain to me what part your family doctor played in your follow‐up.
What role would you like your family doctor to play ?

The interviews were recorded, transcribed verbatim and rendered anonymous. Observations regarding the patients’ behaviour and feelings and their interactions with the interviewer were also recorded.^25^

### 2.2 Data analysis

The interviews were analysed using a thematic approach according to the grounded theory for explanatory purposes.^26^ The interview material was read several times after each transcription. A continuous thematization was performed, which consisted of a list of the topics identified in the course of reading. Low‐level inference was sought to stay as close as possible to the discourse of the interviewee, and interpretation was avoided where feasible. The themes were gradually regrouped and prioritized. Thematic axes emerged from the thematic groupings, and, finally, the major classificatory headings were identified. The analysis was performed independently by two different researchers (JV and PV). Each researcher used the same method to analyse the data. In case of disagreement, the data were discussed with a third researcher (CL). The classification was later analysed independently by a medical anthropologist (CL). Quotes were selected to illustrate the categories and were translated into English by one of the authors (JV). To ensure that the sense of the content was unchanged by translation, the quotes were translated back into French by a native English‐speaking person. The analysis was then submitted to the patients by mail for validation.

### 2.3 Ethics statement

A letter that informed patients about the aims of the study and how it would be conducted and guaranteeing confidentiality, anonymity and the observance of professional secrecy was given to the patients by the GPs or the president of the patients’ association. This letter also explained the way in which consent would be obtained. The patients’ oral consent was requested before and recorded with a digital dictaphone at the beginning of each interview. This procedure of obtaining consent was explained in detail in the form submitted to and approved by the ethics committee of the Clinic Investigation Centres of the Rhône‐Alpes‐Auvergne Area (*Comité Ethique des Centres d'Investigation Clinique de l'Inter‐région Rhône‐Alpes‐Auvergne* [Institutional Review Board Reference Number: 5044]).

## 3 Results

Of the nine GPs who agreed to participate, two did not submit patients because of time constraints (see Table 2). This did not, however, affect the diversity of the sample. Twenty‐three patients were selected and contacted. Two declined to participate because they did not wish to be interviewed at home. Therefore, the analysis involved 21 patients, of whom 16 were recruited from GP practices and five from the patients’ association (see Table 3). Eight were from urban areas, seven from semi‐rural areas and six from rural areas. Thirteen cancers were diagnosed through screening and eight on the basis of symptoms; the time that had elapsed since the completion of treatment ranged from 6 months to 4.9 years. The interviews lasted on average 45 minutes (range: 20‐74 minutes). Data saturation was reached after 18 interviews: three extra interviews were conducted for confirmation. All patients interviewed validated the interpretation of the data and expressed neither disagreement nor the desire to have new elements taken into consideration.

**Table 2:** List of general practitioners (GPs)

	Age	Sex	Type of practice	Place of practice
GP 1	38	F	Group	SR
GP 2	51	F	Group	U
GP 3	45	M	Group	R
GP 4	50	M	Group	SR
GP 5	59	M	Group	SR
GP 6	54	M	Group	R
GP 7	62	F	Individual	U
GP 8^a^	44	F	Group	SR
GP 9^a^	60	M	Individual	U

U, urban; R, rural; SR, semi‐rural.

a GPs who did not include patients.

**Table 3:** Description of participants (N=21)

	Age	Surgery	Radiotherapy	Chemotherapy	Hormonotherapy	Time in years since the end of institutional treatment	Place of residence	Family situation before cancer	Family situation after cancer	Children	Occupation	Sex of attending physician	Number of years of follow‐up by the GP	Type of GP practice
P 1	30	Conservative	+	+	−	1.6	Urban	With a partner	Married	1	Post‐woman	F	8 yr	Group
P 2	63	Conservative	+	+	+	3	Urban	Married	Married	1	Laboratory technician, after the cancer: retired	F	7.5 yr	Individual
P 3	49	Conservative	+	+	+	3.2	Urban	Married	Married	2	Town Hall secretary	F	10 yr	Group
P4	51	Mastectomy+reconstruction	+	+	+	1.8	Semi‐rural	Married	Married	2	Home visit hairdresser, after the cancer: disabled	F	20 yr	Group
P 5	50	Mastectomy+reconstruction	+	+	−	4.8	Semi‐rural	Married	Married	2	Housewife	M	30 yr	Group
P 6	57	Conservative	+	−	+	2.9	Rural	With a partner	With a partner	2	Counter clerk, permanent status after the cancer	M	13 yr	Group
P7	78	Conservative	+	+	+	2.3	Rural	Divorced with a partner	Divorced with a partner	4	Retired domestic worker	M	17 yr	Group
P 8	86	Conservative	+	−	+	4.9	Rural	Married	Widow	2	Retired farmer	M	25 yr	Group
P 9	57	Mastectomy without reconstruction	+	+	+	0.9	Urban	Married	Married	3	Health benefit manager	F	23 yr	Group
P10	52	Mastectomy without reconstruction	+	+	+	2.4	Semi‐rural	Married	Married	1	Teacher	M	12 yr	Group
P 11	61	Conservative	+	−	+	4	Semi‐rural	Married	Married	2	Factory worker	M	10 yr	Group
P12	51	Conservative	+	−	+	0.6	Semi‐rural	Married	Married	3	Retired nursing auxiliary	M	15 yr	Group
P 13	79	Mastectomy without reconstruction	+	+	+	0.3	Urban	Married	Married	1	Retired sales assistant	M	10 yr	Group
P 14	50	Conservative	+	−	+	0.1	Urban	Married	Married	3	Hospital dietitian	F	15 yr	Group
P15	58	Conservative	+	−	+	0.1	Urban	Single	Single	0	Shop manager	F	7 mo	Individual
P16	74	Conservative	+	−	+	1.2	Rural	Married	Married	0	Retired shopkeeper	F	30 yr	Individual
P 17	67	Conservative	+	+	+	1	Rural	Married	Married	2	Retired secretary	F	30 yr	Individual
P18	51	Conservative	+	+	+	3	Semi‐ rural	Married	Married	3	Hospital orderly	M	20 yr	Group
P 19	48	Conservative	+	+	+	1	Semi‐ rural	With a partner	With a partner	3	Bank employee	F	23 yr	Group
P20	54	Conservative	+	−	−	4.2	Urban	With a partner	Single	2	Director of human resources	M	4 yr	Group
P 21	47	Mastectomy+ reconstruction	+	+	−	0.6	Rural	With a partner	With a partner	2	Service agent	M	26 yr	Group

Four main themes emerged from the analysis of the interviews: that breast cancer is a life‐changing event; how patients managed the effects of treatment; how patients viewed the future; and patients’ expectations of their GP.

### 3.1 A life‐changing event

Cancer had permanently altered the lives of all the women interviewed. The patients believed that cancer was a taboo subject, a source of fear in the collective imagination and a possible cause of alienation. Some felt a diminished sense of self because of their disease.

Cancer…it makes you a total misfit… In people's minds there's a kind of deep‐rooted fear.

Patient 5 (P 5, 50 years, mastectomy+reconstruction [mr])

It changed my life completely… it's a complete break… it's not a life, it's survival.

(P 18, 51 years, conservative surgery [cs])

Several patients mentioned a decline in their physical capacities. This debilitating effect led to a loss of autonomy and a sense of isolation.

I've aged… I'm not the same any more… I can't get my work done on my own now… I'm not the same.

(P 7, 78 years, cs)

I can't do anything any more. I never get out… We used to go on trips. I couldn't go away now. I'm all alone.

(P 1, 30 years, cs)

Several patients referred to the breast as a “non‐vital organ.” They had agreed to mastectomy almost with relief, associating the removal of the breast with the disappearance of the cancer. Most of the patients described their illness as an assault on their womanhood and felt that the cancer and its treatment had affected their sex lives. This was felt particularly strongly by young women. Women who had undergone a mastectomy considered the disease to be serious. Younger women had requested the earliest possible reconstruction after mastectomy. Some women described a significant social impact, such as difficulty buying a swimsuit or resuming their professional activities. They confessed to experiencing less sexual desire and satisfaction and difficulty resuming sexual activity. Most of the women were keen, however, to stress that their partner was attentive and understanding. Some reported that their partner was afraid of causing them pain in the area of the breast operated on.

Even after breast reconstruction, I felt that my husband wasn't the same. He turned his head when I undressed and he didn't dare caress me.

(P 4, 51 years, mr)

You feel less of a woman, less fulfilled.

(P 14, 50 years, cs)

Although the women's narratives showed that irreversible changes had occurred in social, professional and personal aspects of their lives, some claimed that cancer had not changed anything. For example, Patient N4, who was aged 51 and who had been a hairdresser before her illness, said that the disease had not changed anything in her life, even when she was she was registered disabled after treatment.

The women rarely mentioned concerns about their body appearance but, when they raised the problem, the GP's advice was appreciated. GPs seemed the best people to confide in about these issues, and the patients wished that they would spontaneously broach these intimate problems.

In contrast to these negative reports, many women said that they had adopted a different approach to life and attained an enhanced awareness of the value of life after their illness. The ordeal of cancer had caused them to develop a concern for other people's welfare. They said they no longer waited to initiate plans. For one patient, the physical and mental suffering wrought by the cancer had given her great strength. For many of the women, the further back in time the treatment, the less it was remembered as a painful event.

I want to do something and I just do it… Life is worth a hundred times more than… any other gift.

(P 4, 51 years, mr)

### 3.2 Managing the effects of treatment

All the women described late adverse effects after treatment. They accepted these effects with resignation as a price that had to be paid for the cure and about which nothing could be done. Most had not spoken on the subject with a health‐care professional. Those who had spoken with a health‐care professional had preferred to do so with a cancer specialist; GPs were sought only rarely. Most of the women said that they had found out what they needed to know by reading, watching television or surfing the Internet. Some attested to the help afforded by patients’ associations, where they could exchange experiences with others in the same situation. Those who were not members regretted not having joined.

The fatigue—it seems that it's normal, then I don't speak about it anymore. It's as the side effects of the hormonotherapy, I've become used to it, I've no choice. But I say to myself that it's not a disaster compared to other ladies…

(P 6, 57 years, cs)

With regard to patients’ associations: ‘They're the only people in a position to really understand, even the tiniest details, even very personal things.

(P 5, 50 years, mr)

Certain patients had tried alternative treatments, such as homeopathy, micro‐physiotherapy and Ayurvedic medicine, in conjunction with standard management. A few women had consulted a psychologist, but only in one case was this on the advice of a GP. Overall, the women regretted not having discussed their psychological distress during appointments with their doctors.

She would tend to say: “Everything all right then?”… “Yes, things are fine.” And that would be it.

(P 2, 63 years, cs)

Some patients had consulted a nutritionist or discovered from books, the media or the Internet about the connection between diet and physical exercise and the prevention of relapses. None had received advice about diet and lifestyle from their GP or from doctors at the cancer centre.

I try to get out and about a bit more than before, because you hear so much about how you should do physical exercise…

(P 19, 48 years, cs)

### 3.3 Their view of the future

At the end of their institutional treatment, many women felt abandoned, as if in a vacuum. Consultations at the cancer centre stopped without an official handover to the GP or another outside doctor.

They send you off, they've done what they had to do… They don't know if you're cured, but they've done the treatment… and after that off you go. You don't get any more phone calls… it's all over. That's what I found the hardest. Even now.

(P 17, 67 years, cs)

Before, I had the impression of living with a sword over my head, but it had a shield that protected me. Since the end of treatment I feel there is more to shield…

(P 21, 47 years, mr)

The women said that they felt that they no longer had the support of those around them, who considered that the end of treatment meant a return to life as it was before. Most said they went through a phase of coming to terms with a changed body and a new existence.

At home… I had support… but from the day it was finished, that was it … I'm not allowed to talk about it anymore… When I'm tired, they don't understand… For them it's a thing of the past.

(P 3, 49 years, cs)

All of the women said that they lived with a feeling of uncertainty caused by the fear of relapse. This feeling was exacerbated by rounds of examinations and follow‐up visits. Paradoxically, most patients were in favour of complementary examinations, because they provided reassurance. They would have liked to hear the word “cure,” but their doctors spoke only of “remission.” The cancer expert seemed to them to be the person best able to give them reassurance. They felt the need for straight talking in their conversations with health‐care professionals. The slightest symptom reawakened the fear of relapse. They were also worried about what might happen to their daughters. For many patients, the GP was the doctor who explained the results of follow‐up examinations and provided answers to their family's queries. One patient sensed awkwardness in the manner of her GP when follow‐up laboratory results were abnormal. The GPs’ ability to listen and give their time contrasted sharply with the rapid pace of specialist consultations.

The return to work was described as sometimes difficult and full of uncertainty. One patient commented on the lack of understanding among her colleagues. The women were aware of the important role played by the GP, sometimes in collaboration with occupational health‐care professionals, in easing their return to work.

However things are there's always this little threat hanging over you… when there's something wrong, that's immediately what you think of.

(P 10, 52 years, mastectomy [m])

For the moment, you are in… remission… but they never say: you're cured.

(P 17, 67 years, cs)

The women considered themselves lucky in comparison with patients whose cancer had undergone an unfavourable evolution. They felt generally optimistic and hopeful. Several patients said that they had received great help from their circle of family and friends, both in the workplace and in community groups. This was particularly true for members of the patients’ association, who benefitted from sharing their experiences with individuals other than doctors.

### 3.4 What they perceived from their primary care physician

Most of the patients believed that the GP had a key role in centralizing the different elements of their medical file. For most patients, it was during their illness that the notion of a family doctor took on its full meaning. They appreciated their close relationship with their GP, who knew and cared for the whole family and was therefore able to give support to all its members. The feeling of trust was all the greater because their relationship was long‐standing.

She was affected by it. When I had the operation, she called me at the hospital. I know I can count on her. And she's very close to my daughters.

(P 19, 48 years, cs)

If the children wanted to call the doctor to ask a question, well they could.

(P 18, 51 years, cs)

Post‐treatment follow‐up care was coordinated in most cases by the cancer centre. Certain patients regretted that they were not more closely overseen and were incapable of naming the doctor incharge of their care. In general, the women had greater confidence in the cancer specialist but would sometimes visit their GP. Most survivors thought that the GP should be responsible for centralizing the many elements of their medical records. They agreed to be followed up by their GP, provided that there was close co‐operation with an oncologist. They believed that this co‐operation should allow the GP to book an appointment with a specialist more quickly in case of problems. Women who lived far from the cancer centre wanted their GP to have a greater role in their post‐treatment follow‐up, to avoid long trips.

With the specialists you get the distinct impression that they don't listen to you in the same way as the GP. When I do not understand or when I have a problem, I can always call him.

(P 15, 58 years, cs)

Nowadays supervision is in the hands of the specialist. Which is reassuring because the cancer doctor knows his job.

(P 10, 52 years, m)

I don't know who to turn to… I don't know if she's qualified enough, competent… A family doctor doesn't look after a cancer… I may be wrong.

(P 3, 49 years, cs)

The women also called on the GP to renew prescriptions made out by the cancer centre (for physiotherapy or medication) or to obtain administrative documents (sick leave certificates, applications for spa treatments, disability allowance forms).

## 4 Discussion

### 4.1 Principal findings

This is the first French study to analyse the experiences and perceptions of breast cancer survivors. The women who participated in our study experienced breast cancer as an irreparable break in their lives. The end of treatment was not felt to be the end of the cancer. The difficulties encountered included an undermining of their identity as a woman, physical changes, an altered sex life and a diminished sense of self‐worth. Their lives were permanently marked by uncertainty and anxiety about the future because of the fear of relapse. They felt abandoned at the end of treatment. Among their health‐care professionals, the GP and cancer expert were perceived as the best suited to give reassurance by providing technical expertise and personal empathy, respectively. The women wanted well‐coordinated care providers to monitor them. They regretted the lack of discussion about their psychological and sexual difficulties and thought that their GP should ask them about these issues. They wanted their GP to be more involved in their post‐treatment follow‐up on the condition of good coordination between the GP and the oncologist.

### 4.2 Results in the context of other studies

Breast cancer has an intimidating reputation as a disease because of the symbolic importance of the breast and the mutilating effects of treatment. The breast represents many things: gentleness, motherhood, sexuality, sensuality and safety.^23^, ^24^ In a review of the literature, Lewis et al.^27^ encountered the same difficulties as those described by our patients. Women everywhere would like the psychological problems arising from breast cancer to be taken into account. Advice and help are provided in the professional recommendations for breast cancer follow‐up care in France^10^ and North America,^11^, ^24^ and in the French Cancer Plan II 2009‐2013.^12^

Studies on the post‐treatment follow‐up and management of cancer have mainly concentrated on the indications for and benefits of complementary examinations.^28^, ^29^ Breast cancer has become a curable disease that, like other chronic illnesses, requires the organization of long‐term care. Follow‐up is focused on screening for recurrence, which is of concern to survivors and physicians alike.^28^ Currently, specialized centres have to cope with the follow‐up care of ever‐increasing numbers of patients.

In our study, as in that of Wright et al.,^30^ women consider their confidence in the ability of the doctor incharge of their care most important. They expected a personalized relationship. GPs have a holistic approach to their patients, which is of particular value in the management of chronic diseases. However, our study showed that GPs were not considered by their patients to be fully qualified to conduct the follow‐up care of breast cancer, which was believed to fall outside their remit. As in other studies, the patients had greater confidence in cancer specialists but, simultaneously, found them unreceptive.^15^, ^16^, ^17^

Two randomized controlled clinical trials^31^, ^32^ that compared follow‐up by GPs and cancer specialists showed no differences in time to diagnosis, levels of anxiety or quality of life between the patient groups. Patients in groups followed up by GPs expressed greater satisfaction; furthermore, an economic analysis showed that the cost of follow‐up care performed by GPs can be lower than that of follow‐up care performed in hospital.^33^

Therefore, primary health‐care professionals can readily provide follow‐up care for these patients. In France, the Cancer Plan II 2009‐2013 aimed to give greater scope to GPs for the treatment and personalized follow‐up of patients. Innovative, shared follow‐up schemes allow a two‐way exchange of information and create ties between GPs and specialist centres.^34^ These initiatives may foster greater patient trust; however, in a recent controlled trial of an attempt to increase the involvement of GPs in cancer rehabilitation, this aim was not achieved.^35^ Further studies must determine how GPs can be involved in the management of cancer treatment and post‐treatment care.

### 4.3 Limitations and strengths

Careful selection of a varied panel of women increased the external validity of the study and allowed collection of a broad data spectrum and data saturation. The sample of survivors was diverse in terms of age, treatment type, geographical location, family situation and socio‐economic status. However, it was not a representative sample, because the study was conducted in a predominantly rural region of France. Some GPs may have unconsciously selected patients with whom they had a good relationship. To influence the women's replies as little as possible, especially with regard to questions concerning the attending physician, the research interviewer was a medical student. That the researcher was a woman, that she had no connection to the attending physician, that there were no observers and that the surroundings in which the interview took place were familiar all helped to establish the patients’ confidence.

The interviews were fully transcribed by the person who conducted them in the interests of objectivity and reproducibility and to avoid subjective interpretation of the data. We did not use triangulation for data collection. A focus group approach did not seem appropriate for the analysis of patients’ mental distress and private lives. The triangulation used for the analysis and the validation of the data by the participants enhanced the internal validity of the study. The participation of three researchers limited personal interpretation. A matrix analysis could have been used to identify characteristics of the patients that may have influenced their perceptions.

### 4.4 Perspectives

The findings of this study show that breast cancer survivors expect better collaboration between cancer specialists and GPs during their post‐treatment follow‐up. Women with breast cancer have a complex care path involving multiple health‐care professionals. Expansion of the role of GPs in this health‐care path requires their involvement from the acute phase of treatment onwards. Patients must feel that GPs are familiar with their medical records and that they are working with the specialist centre. The exchange of information between the cancer centre and the GP is essential from the time of diagnosis to the time of post‐treatment care and should occur through care networks. It is therefore necessary to provide more resources for outpatients care. Further qualitative studies must explore both the perceptions of GPs regarding their involvement in monitoring cancer survivors and the perceptions of cancer experts on how to work with GPs. Subsequently, quantitative studies are necessary to evaluate the efficacy of this division of tasks in the French health‐care system.

## 5 Conclusion

Breast cancer leaves an indelible mark on the life of a woman: it is experienced as an assault on her womanhood and changes her perception of the future. The end of treatment is not perceived as the end of the cancer. Although certain difficulties in life after cancer are common to all patients, each experience is unique.

When hospital follow‐up is complete, French GPs do not have a clearly defined role in the post‐treatment management of breast cancer. Nevertheless, they have the closest contact with survivors of the condition and centralize all elements of their medical files. Although women have more confidence in the ability of oncologists, they are not opposed to the participation of their GP in follow‐up care, provided that there is full collaboration with the oncologist. They desire to be listened to and supported. Implementation of the French Cancer Plan II 2009‐2013 and its guidelines may provide an opportunity for closer collaboration between GPs and oncologists and lead to the sharing of information and tasks, resulting in effective and individualized follow‐up care.

## Conflict of Interest

The authors declare that they have no conflict of interest concerning this article.
